# Expectation and Satisfaction with Nursing Care among Hypertensives Receiving Care at a Resource-Constrained Hospital in Ghana

**DOI:** 10.1155/2020/6094712

**Published:** 2020-03-07

**Authors:** Kennedy Dodam Konlan, Mavis Armah-Mensah, Rita Aryee, Theresa Akua Appiah

**Affiliations:** ^1^Department of Social & Behavioural Sciences, School of Public Health, University of Ghana, Accra, Ghana; ^2^Training & Research Unit, Nursing Directorate, Korle-Bu Teaching Hospital, Accra, Ghana; ^3^School of Business, Ghana Institute of Management & Public Administration (GIMPA), Accra, Ghana

## Abstract

**Background:**

Hypertension accounts for a third of the global preventable premature deaths. In Sub-Saharan Africa, hypertension is the most rapidly increasing cardiovascular disease (CVD) and the second leading cause of death. Proper management of hypertension requires adherence to management by patients and this is partly possible if patients feel satisfied with the nursing care they receive. Satisfaction with nursing care is only possible if there is a congruence between the expectations of care and the actual care received from nurses.

**Aim:**

We explored the expectations and satisfaction of Ghanaian hypertensives with nursing care received at the Korle-Bu Teaching Hospital (KBTH).

**Methods:**

In this qualitative study, a phenomenological approach was used to gather data about the lived experiences of patients with hypertension about nursing care. In-depth interviews (IDIs) were conducted among sixteen (16) patients with hypertension from the hypertensive Out-Patient Department (OPD) Clinics of the Medical Department at the KBTH. Only patients with history of previous admission(s) at the KBTH during the immediate past six months were purposively recruited. The respondents were interviewed about the nursing care received during their immediate past admission(s) at the KBTH using an IDI-guide. The IDIs were recorded digitally, transcribed verbatim, and reviewed severally and thematic analysis was done. Nvivo 11 software was used to manage the data and aid with the thematic analysis.

**Results:**

The results of this study showed that Ghanaian hypertensive patients perceived nurses as key players in the management of patients. On the respondents' expectations from nurses prior to their immediate past admissions at the KBTH, the data revealed the responsiveness of nurses to patient needs, prompt pain management, high confidentiality level of nurses, rendering of efficient health education, maintenance of therapeutic work environment, and ensuring effective communication as well as professional/ethical practice from the nurses. On the question of what made nursing care satisfying, it was observed from the respondents that they considered the competence of nurses, maintenance of therapeutic environment, and also effective handling of confidential information as determinants of their satisfaction with nursing care. Further, the respondents identified some key areas of dissatisfaction and these included the responsiveness of nurses to patient needs, prompt pain management, effectiveness of health education, and provision of culturally sensitive communication. Disproportionate distribution of nursing staff across the three nursing shifts, unethical practice among some nurses, inadequate resources for work, and low work morale of some nurses were identified as factors responsible for the gaps between patient expectations and actual care received.

**Conclusion:**

Our study concludes that continuous professional development programs for nurses should focus on the areas of dissatisfaction so as to improve care for hypertensives. We also recommend that nursing staff distribution across the various shifts should be of keen interest to nurse managers if hypertension care in particular and overall patient care in general are to improve.

## 1. Background

Currently, noncommunicable diseases (NCDs) like metabolic syndrome (MetS) are said to be rising alarmingly in the Ghanaian population particularly among females [1, 2] and there are fears that this could worsen in the not-too-distant future because current data is suggestive that young adults with parental history of hypertension have increased risk for MetS [2]. Nurses are influential members of the healthcare team and could play a critical role reversing this rising burden of NCDs [3]. Hypertension, a common NCD, characterised by sustained high blood pressure of above 140/90 mmHg in an individual after three measures [2, 4] is now a global epidemic and nurses are crucial staff needed to confront this epidemic as well as its consequences [3, 5]. Proper management of hypertension requires adherence to management by patients [2, 4] and this is partly possible if they are satisfied with the nursing care they receive in hospitals [6]. There is a general consensus on the significance of nursing interventions in shaping the patients' total satisfaction with the health services they receive [3, 6] and they may influence patients' compliance with therapy particularly chronic-diseased patients [4]. Further, the awareness about the needs and expectations of patients with hypertension is important in improving the quality of the nursing services they are provided in hospitals [6, 7].

Patient satisfaction with nursing care was conceptualized by Risser [7, 8] as the degree of congruence between patients' expectations of ideal nursing care and his/her perception of the real nursing care he/she receives [8]. Most definitions have certain elements of subjectivity including one definition which states that it is a measure of a patient's or a family's opinion of the care received from the nursing staff [9]. It is envisaged that timely, accessible, appropriate nursing interventions and continuous and effective nursing service delivery are important components of healthcare quality [10]. It is no wonder that states, international organizations, corporate bodies, and even individuals are working tirelessly to promote quality care and patients' protection and safety from patients' perspective [7, 10]. In fact, it has been suggested the extent to which healthcare users are satisfied with their nurses may be a key factor underpinning their health behaviour and healthcare utilization [7]. It has been established that patients' satisfaction with nursing services is particularly important since the nursing staff constitute the majority of health professionals and are constantly by the patients' side in order to satisfy patients' needs [10, 11].

Hypertension is now classified as a public health emergency in Sub-Saharan Africa (SSA) [2, 5] and it is the leading risk factor for cardiovascular morbidity and mortality [1, 2]. In Ghana, hypertension accounts for 4% of all Out-Patient Department (OPD) attendance and 4.6% of all deaths [12]. At the KBTH, noncommunicable diseases (NCDs) continue to lead the top ten (10) causes of OPD attendance [13]. Hypertension and hypertension-related morbidities account for almost 35% of cases seen at the KBTH [13]. This high number of hypertension-related cases puts considerable pressure on nurses as these conditions require life-long management [2]. This is occurring against the backdrop of the persistent complaint from the management of the KBTH about inadequate nursing staff and this has the potential of predisposing the current nurses at the KBTH to high workload [13]. High workload among nurses is known to carry the risk of burnout syndrome [14] which could have negative consequences for recipients of nursing care [10].

## 2. Aim

We explored the expectations and satisfaction of Ghanaian hypertensives with nursing care at the Korle-Bu Teaching Hospital.

### 2.1. Method

#### 2.1.1. Study Design

We chose a descriptive qualitative approach for this study in order to obtain in-depth data [15]. The theoretical model underpinning the study was the Donabedian quality assurance framework [16]. The Donabedian quality assurance framework provides that relevant elements of care satisfaction may be categorized according to whether they are related to structure (facilities, personnel), process (technical process, interpersonal process), or outcomes (somatic, psychosocial, and financial) of care [17]. Thus, we sought to determine how structural, process-related factors or outcomes could influence satisfaction with nursing care among hypertensives. In this study, the phenomenology strategy was used to gain in-depth information [18] about the lived experiences of hypertensives about the nursing care received during the immediate past admission at the KBTH. “Phenomenology in qualitative research allows participants to share their perceptions, feelings, and lived experiences in a given situation and how these experiences affect their views about a given situation” [15, 18]. The philosophical approach that was chosen for this study was interpretivism. Interpretivism perceives that social events are created from perceptions of social actors [18, 19]. Some of the qualitative approaches associated with the interpretivist paradigm include focus group discussions, in-depth interviews, ethnographic case studies, among others [15, 19].

#### 2.1.2. Setting

The study was conducted at the Korle-Bu Teaching Hospital (KBTH), a resource-constrained health facility in Ghana. The KBTH is located in the southern part of Ghana and it is the national referral hospital in Ghana. It is the third largest hospital in Africa [13]. The KBTH which was established in 1923 has grown from an initial 200 beds to 2000 beds and 17 clinical and diagnostic departments. The hospital is traditionally known to receive huge numbers of referral cases from across the country with daily average patient attendance of 1,500 with average daily admission of 250 patients [13]. The respondents were selected from the Hypertensive OPD Clinics of the Medical Department at the KBTH. These clinics run for four days in a week from 8am to 2pm. The working days for the clinics were Mondays, Tuesdays, Thursdays, and Fridays.

#### 2.1.3. Selection of Participants and Data Collection

We included male and female hypertensive patients with previous history of admission at the KBTH within the immediate past six months who were now visiting the Hypertensive OPD Clinics of the Medical Department at the KBTH for review after discharge. Purposive sampling technique was used to select the respondents for the study. The inclusion criteria for the selection of the study participants included hypertensive patients between the ages of 35 and 65 years, history of previous admission with hypertension within the immediate past six months, and being well oriented to time and place with no signs of mental illness. We interviewed sixteen (16) respondents and each interview lasted between 30 and 45 minutes. All interviews were conducted in English or Ga or Twi, after which the data was transcribed. The Twi and Ga audio files were translated before transcription. Thus, patients who could not speak Ga or Twi or English were not included in the study.

Data collection was done with the aid of a tape recorder and an in-depth interview (IDI)-guide after obtaining voluntary written consent from each respondent. The IDI-guide was designed in English and translated into the two commonly spoken Ghanaian local languages, Twi and Ga, where necessary during the data collection. The IDI-guide was unstructured and consisted of open-ended questions about expectations about nursing care prior to immediate past admission at the KBTH, satisfaction with nursing care during the immediate past admission, and factors accounting for expectations about nursing care not being met during the immediate past admission at the KBTH. The data collection period was from October to November, 2017. The researchers maintained a nonjudgmental approach throughout the data collection.

#### 2.1.4. Data Analysis

Huge data set is usually obtained when an investigator decides to use the phenomenological approach in any study [15, 16]. This qualitative data set normally comes as audio files of tape recordings, interview notes, and field notes which are often analyzed together to give meaning to the social construct of interest [15–17]. Thus, the IDIs in this study were recorded digitally with an audio recorder after seeking the respondents' permission and transcribed verbatim. All the transcripts were anonymized and no identities of the respondents were included in the notes. Each participant was given a unique identification number beginning with R meaning respondents followed by the number of the respondents as per their sequence during the interview. Hence all the transcripts were numbered from R1 to 16. An independent person at the Training and Research Unit of the Nursing Directorate at the KBTH reviewed all transcripts to ensure they reflected the views in the audio files/recordings. The transcripts were then entered into a word processor and imported into Nvivo 11.

Thematic analysis was employed so as to give meaning to the data as recommended for most qualitative studies [15, 16]. “Guest, Macqueen and Namey, summarizes the process of thematic analysis as consisting of reading through textual data, identifying themes in the data, coding those themes, and then interpreting the structure and content of the themes” [15, 18]. Based on the above, we developed a codebook first and went further to create nodes within NVivo 11 using the developed codebook. Then, we proceeded with line-by-line coding of the various transcripts. We had two of investigators doing the coding and coding comparison.

#### 2.1.5. Rigor

Trustworthiness of the study was ensured by member checking from the participants at Hypertensive OPD Clinics of the Medical Department at the KBTH during the concurrent data analysis and it facilitated the full understanding of the participants. Also, writing detailed field notes and discussion of findings among the investigators helped to ensure trustworthiness of the study.

#### 2.1.6. Validity and Reliability

Member checking was done by verifying from the respondents the data generated to maintain confirmability and credibility [18]. In order to ensure dependability, all respondents were interviewed using the same IDI-guide. Peer briefing was done among the investigators to ensure all aspects of the data were covered during the thematic analysis. Further, detailed description of the protocol was done in order to ensure replicability of the study [15, 18].

#### 2.1.7. Ethical Issues

The study was approved by the Scientific and Technical Committee as well as the Institutional Review Board of the Korle-Bu Teaching Hospital (Protocol Identification Number: KBTH-IRB /00017/2017). Written voluntary informed consent was obtained from all participants and permission was obtained for audio-taping of IDIs. Also, permission was sought from the Deputy Director of Nursing Services at the Central OPD and the Medical Department of the KBTH prior to data collection. The researchers also maintained the confidentiality of the respondents throughout the data collection by assigning specific codes for each respondent. The authors ensured anonymity of respondents by representing interview transcripts of the respondents with R1, R2, R3, …, R16. The audios and transcripts were placed under lock in a cabinet for research purposes at the Training and Research Unit of the Nursing Directorate at the KBTH with the key available to only the investigators. Also, respondents were further assured that they could opt out of the study at any time without any effect on their care.

## 3. Results

The data was organized concurrently during the data collection by applying the process of thematic analysis. The themes that emerged from the data were the views of the respondents on the nursing care at the KBTH. During thematic analysis, the investigators are supposed to remain faithful to the respondents by using what is known as bracketing [15, 18]. “Bracketing is used to reduce personal biases in presenting the findings of a research” [15]. Hence, we summarized the results of our study using the themes that emerged from the data and supported by the narratives with illustrative quotes from respondents as required in phenomenology [18]. The respondents were numbered (R1–R16) and the number after each quotation shows which of the respondents was talking.

### 3.1. Expectations of Clients with Nursing Care before Arrival in the Hospital

When the respondents were asked about their expectations of nursing care, the responses were categorized and after open coding of the data, the researchers identified seven themes as the respondents' expectations of nursing care. These themes were responsiveness of nurses to patient's needs, prompt pain management, high confidentiality level of nurses, sufficient health education, ensuring satisfactory work environment, effective communication, and competence in general nursing work.

On the theme of nurses being responsive to their needs and pain concerns, the respondents reported that, prior to coming to the hospital and in fact coming into contact with nurses, they expected them to be very responsive to their needs and to act quickly in meeting those needs. They also claim that they expected nurses to treat pain as an emergency. They also expected them to console them when they were in pain.

“I expected that I would be attended to appropriately by nurses and my pain taken care of appropriately, nurses are always around us so I expect them to do more when am in pain” (R8).

“While waiting to see the doctor, I expect nurses take care of any pain I have, they are always with us” (R5).

“Nurses should help me get what I need, if am in pain, I believe it's their responsibility” (R14).

“The nurses need to attend quickly to us when we ask for something and not delay, when we are feeling pain, they have to be quick to give us our medications” (R3).

“The nurses should try to attend to our pain early and respond quickly when we need something since we are sick” (R15).

On the confidentiality level of nurses, it emerged from the respondents' narratives that they expected nurses to keep their information private and not broadcast it anyhow as some of such information was seen to be sensitive to them.

“Since Korle-Bu is a big place with so many people; I expect my information to be kept secret” (R13).

“Nurses have access to my private information so I expect them to be secretive” (R16).

“I expect nurses to keep our information secret” (R12).

“I expect the nurses not to share our information with other people” (R7).

“The nurses should try to keep our information private” (R6).

“In a big place like this, I think the nurses have to try to keep our private information secret between themselves” (R9).

The respondents claim they expected sufficient health education from nurses as most of them claimed nurses needed to educate them.

“The hospital is not my work place, so I expect nurses to talk to me on my condition” (R4).

“I have not gone to school, so I expected nurses who should know better and should talk to me on what is wrong with me” (R7).

“I expected that they should try to tell us what is happening/wrong with us because they are in the hospital and they know why some things are happening” (R1).

“as for me my expectation was that the nurses talk to me about what is wrong with me in simple manner” (R8).

“my expectation is that the nurses would teach me what to do and not to do so that i can get well” (R6).

Also, the respondents stated that they expected nurses to maintain a good working environment and communicate effectively with them as clients. They further expected a humane communication with mutual respect between the nurses and them, the recipients of care.

“I expected the nurses to make sure the place is clean and they must learn to talk to us at least we are also human beings” (R7).

“my expectation was that nurses are always with us on the ward so they should do well to ensure the cleaners do their work” (R12).

“I only expect nurses to talk to me and my relatives with dignity and respect because it is not a curse to be in the hospital” (R9).

“I expected the nurses would learn to be nice and smile towards us” (R4).

“I expected the nurses to talk nicely to us so that we too will be happy” (R1).

“The nurses have to make sure the place is smelling nicely and ready for work” (R2).

On the competence of nurses, the respondents claimed they expected nurses to assist them get well. Most of them said they expected nurses to help them see their doctors and to perform their work very well according to their training.

“I expected the nurses to assist me see the doctor so that I can go home early and do my work‥‥. I don't want to delay/waste my time here” (R1).

“my expectation is for the nurses here to know their work, I expect them to be different and highly skillful” (R2).

“I expected the nurses to work hard and remain as professionals” (R6).

### 3.2. Aspects of Nursing Care That Respondents Seemed Satisfied or Dissatisfied

After open coding of the data on aspects of nursing care that the respondents were satisfied or dissatisfied with as per their expectations prior to coming to the KBTH, the following was found:

On the responsiveness of nurses to respondents' needs and prompt pain management, it emerged from the respondents' narratives that nurses respond slowly to their needs with most of them ignoring their needs. They also claim pain management was very poor and that they had to answer several questions on whether they were really in pain before being given medication. The respondents also claimed nurses were not also compassionate enough, with most of them claiming the nurses did not really show the compassion expected of them as nurses. Some claim the nurses also did not pay attention to their mental/psychological pain especially for those with life-threatening diagnosis.

“You never get help with most of the things unless they see you as well to do or educated or if you give them tips/gifts” (R1).

“They are mostly friendly, kind and nice to only their friends and their relations or Church members, most of the young ones are always on their phones and seem not to care about you and they waste time when you request for something” (R11).

“They care less about our needs and they take so long before responding to your request” (R3).

“They think we are dramatizing when we say we are in pain so they don't give us the drugs ooo” (R2).

“Sometimes they won't even mind you when you ask for something especially the small ones”(R14).

“They come to only give you pain medication if they see that you are making so much noise” (R3).

“Even if they have drugs for pain, they delay before giving it” (R6).

On the confidentiality level of the nurses, the results indicated a general good confidentiality level of nurses. Most respondents claim nurses kept their problems and conditions well and private. Thus, the respondents were generally satisfied with the levels of confidentiality being maintained by nurses.

“The nurses here try paaa, they keep our information secret” (R8).

“I have not heard them talking about my things to any one” (R13).

“The nurses here are really secretive, they don't share our information” (R3).

“The nurses are good when it comes to keeping our information” (R2).

“Most of the nurses are really wonderful when it comes to keeping our information” (R8).

“The nurses here are people you can trust” (R5).

On the sufficiency of the health education received from the nurses, the respondents claim nurses did not do well with the health education given to them. The transcripts from the interviews indicated that nurses provided little or no health education and mostly did not have any time allotted to provide education to respondents.

“The nurses never spoke to me about my condition” (R1).

“The nurses don't have time to talk to us about our condition” (R2).

“The staff have never spoken to me about my disease, only once that they told me about the drugs” (R12).

“The nurses don't teach us here, they give us only our drugs” (R10).

“The place is full of people so they don't educate us” (R4).

“Sometimes they talk to us small about our condition but not so detailed” (R16).

“The nurses are too busy to teach us but sometimes the students do” (R6).

On the theme of maintaining a satisfactory work environment and therapeutic communication, the interview transcripts revealed that the respondents were generally satisfied with the work environment.

“The place is neat” (R1).

“The place is looking better than I thought of Korle Bu. now Korle bu is good” (R3). “I think the nurses are doing well” (R4).

“The place is well arranged and fine” (R5).

However, there was gross dissatisfaction of respondents with the communication skills of nurses; most of them claim nurses lacked the key communication skills needed for their work. Most claim nurses spoke harshly to them or other clients and never showed any signs of remorse.

“They keep talking very loudly to you as if you have offended them” (R6).

“The nurses are simply not good when talking to even us the elderly, being sick is not a curse or a crime, the shouting and disrespect is too much” (R5).

“I think the nurses need to learn how to talk” (R12).

“The nurses do not really do not behave as if they have being trained well on how to talk to patients” (R15).

On the competency level of nurses, the transcript revealed that nurses were very competent in doing their nursing work. However, most of the respondents only stated that their nurses were competent but when asked about the specific nursing care, they could not say. They mostly saw the nurses as an assisting tool of the physician/doctor.

“Nurses are supposed to help me see the doctor” (R13).

“Nurses help clients see the doctors and give injections and drugs on the ward, I think they are doing well” (R14).

“The nurses here are good at what they are doing” (R7).

### 3.3. Possible Causes of Why the Expectation of Nursing Care Was Not Met

On the factors harbouring the achievement of expected nursing care, the transcripts from the IDIs revealed that the factors below were responsible. Disproportionate distribution of staff for the various nursing shifts accounted for the respondents' expectations of nursing care not being met. Most of the nurses reported for morning shifts with few staff reporting for afternoon and night duties during their stay on the ward. This disproportionate distribution of the staff resulted in high workload for the night nurses. The workload on night nurses was one factor that led to the widened gap between respondents' expectations of nursing care and the actual nursing care rendered. The respondents claim that the workload on night and afternoon nurses was overwhelming and made it practically impossible for those nurses to render the supposed care they needed or should have received.

“the hospital has so many patients but the staff are few especially during the night, so they can't do what they should be doing” (R10).

“Nurses have a lot to do and even when they try their best to help, their work seem to be too much especially the night nurses. Most of the nurses come for morning duty with few reporting for night” (R14).

“They never get time to even relax; they have to run up and down especially when someone becomes an emergency and yet they are few at night‥‥.” (R16).

“it seems most of them come for the morning and afternoon time with only few at the night duty, this actually burdens the night staff” (R9).

“How can you expect only three nurses to come for night and do all the work” (R3).

Low morale for nursing was identified as another factor responsible for why expectations could not be met. A good number of the respondents claimed some nurses had no motivation for getting into nursing or lost their interest in nursing and hence never acted professionally.

“They seem not to like what they are doing, so they just do it anyhow” (R11).

“The young ladies just don't smile when they are even attending to you, they feel like finishing quickly and going away, they don't care how they do it” (R13).

“Most people are in hospital work/nursing because of money so they even forget that we the patients have needs to be met‥‥‥.‥‥‥‥‥hmmmm, it's a problem ooo” (R7). “It is like the nurses have lost the interest in nursing so they don't care” (R9).

Inadequate working equipment/low resources for work was identified as another factor accounting for respondents' expectations not being met. Respondents claim they observed nurses complaining about the absence of one thing or another and that eventually affected the care they rendered.

“sometimes we don't get the drugs from the hospital pharmacy and what can the nurses do‥‥. so I will say its not their fault” (R2).

“Sometimes it's not the nurses, they might want to help you but they don't have the things to work with” (R6).

“At times, they don't have the things to work with” (R12).

Age of the respondents was identified as being responsible for the contrast between respondents' expectations with nursing care and their satisfaction with care; respondents claim they expected that the respect from home accorded to them be given to them by nurses and this was nonexistent.

“The young people like complaining about the nurses, we the old ones don't care much; we see them as our children” (R5).

“We the older clients tend to have less satisfaction with nursing care than younger clients” (R6).

Noninvolvement of the respondents in care was identified as yet another factor causing the expectations of nursing care not being met. They stated that the nurses sometimes did not involve them and their relatives in care and hence they did not even understand what the nurses were doing and hence could not appreciate it or help.

“They just keep doing their things without even minding us, we don't even know what they are doing and they don't even bother to involve me or my wife” (R12).

“Nurses sometimes think they know best so they don't even involve us, I just don't understand” (R8).

Prolonged stay in the hospital was also identified as one of the factors responsible for the respondents' expectations not being met. According to them, overfamiliarity made the environment boring, and prolonged stay made the nurses not concentrate on them as they had to attend to new clients with priority.

“When you stay for too long, sometimes they ignore you and complain that you are worrying them” (R1).

“Sometimes the environment is too boring for me and makes me not to appreciate the care I even get” (R8).

“its like they are angry, I stayed for long so they don't care like when I came earlier” (R12).

## 4. Conclusion

The results of this study showed that Ghanaian hypertensive patients perceived nurses as key players in the management of patients. On the respondents' expectations from nurses prior to their immediate past admissions at the KBTH, the data revealed the responsiveness of nurses to patient needs, prompt pain management, high confidentiality level of nurses, rendering of efficient health education, maintenance of therapeutic work environment, and ensuring effective communication as well as professional/ethical practice from the nurses.

On the question of what made nursing care satisfying, it was observed from the respondents that they considered the competence of nurses, maintenance of therapeutic environment, and also effective handling of confidential information as determinants of their satisfaction with nursing care. Further, the respondents identified some key areas of dissatisfaction and these included the responsiveness of nurses to patient needs, prompt pain management, effectiveness of health education, and provision of culturally sensitive communication. Disproportionate distribution of nursing staff across the three nursing shifts, unethical practice among some nurses, inadequate resources for work, and low work morale of some nurses were identified as factors responsible for the gaps between patient expectations and actual care received.

## 5. Discussion

The study found an effective collaborative engagement between nurses and patients in which nursing care results in a sense of patients wellbeing which could be described as being free from the danger of hypertension-related complications and empowered to thrive by themselves [19, 20]. Thus, this overarching structure drives the patients' desire to have their expectations for specific needs being met, reducing pain, provision of effective and culturally sensitive communication, and health education. It appears from the data therefore that a collaborative engagement with the patient as a whole and ensuring they flourish in the midst of the restrictions of their ailments is the basis of a satisfying nursing practice from the perspective of the patient.

The results of the study showed that the respondents were mostly satisfied with the technical aspect of care and less with hotel services and that seems to be in line with the literature. Many researchers have found high satisfaction with the technical aspect of nursing care [19]. Respondents' satisfaction with the technical aspect of nursing care was highly ranked and that can be partly attributed to the great emphasis given by the working system to the technical aspect of care [19]. Evidence of satisfaction with the technical care and less with the information received is also reported in several European studies [20]. The implementation of the mechanistic working model leaves nurses with no room to develop an interpersonal aspect of care [19, 21].

### 5.1. Expectations of Clients with Nursing Care before Arrival in the Hospital

On the responsiveness level of nurses, the data showed that respondents expected nurses to respond appropriately to their needs. The respondents also preferred prompt pain management when they were in pain. These findings are in line with the findings of [21] where it is argued that a basic expectation among hospital patients is prompt responsiveness of nurses to their needs, proper pain management, and assurance that they will be attended to by skilled and competent staff that will treat them professionally and efficiently and also with those by [19] in a study on patient's perception of nursing care in a large teaching hospital in India, where more than 95% of the patients had good expectation of “responsiveness,” “availability,” and “ward organization” as well as technical capability of the nurse [9, 20].

The findings on the confidentiality level of nurses revealed that respondents expected nurses to treat them with high level of privacy and to keep their information in confidence. These findings are in line with those identified by [10] in a study at a referral hospital in Alberta where patients said they expected to be treated as unique individuals with needs which required prompt attention and be known by more than a diagnosis and treated as a person.

The results also showed that respondents expected nurses to give them sufficient health education. The respondents said they expected nurses to give them simple explanations in a language they could understand on their conditions and options. These findings are in line with those by [21] involving 200 patients on their expectations with nursing care in the context of healthcare in a general hospital in Argentina; the researchers observed that 84% of patients claim that nurses needed to provide them with an effective health education regarding their conditions, but the findings are in contrast with those identified by [8, 19] where patients claimed nurses were to assist doctors translate health information for them and had no responsibility of giving health education.

The respondents also stated that they expected nurses to provide a clean work environment for their recovery. They also stated they expected nurses to provide effective communication. They expected nurses to be humane and treat them with dignity and respect. These findings are in line with those expressed by [10] in a study on patient's perception of nursing care in a large teaching hospital in India, where patients said they expected therapeutic and culturally appropriate communication with patients.

Respondents also stated they expected nurses to be professional in their dealings with clients and to deliver quality nursing care. These findings are in line with those expressed by [3] where patients stated they expected nurses to have a command of specific knowledge about each patient and his treatment. The finding is also in line with those expressed by [10] where clients point out that they expect to encounter with nursing staff that are proficient, professional, and knowledgeable to enhance their satisfaction with nursing care. The findings are also in line with those expressed by [19] that patients feel that their body is in safe hands if nurses are competent and skillful; and competence gives them a sense that the staff knows what they are doing.

### 5.2. Aspects of Nursing Care That the Patients Were Satisfied or Dissatisfied

On the competence level and professional attitude of nurses, the study revealed that nurses were generally competent in nursing care and adhered to professional practice with respondents expressing satisfaction in these areas. This finding was in contrast with those identified by [7, 20] where clients reported incidents of nursing care providers being “unprofessional,” “incompetent,” “rude,” and “snotty,” nurses who “blew patients off,” and staff who were “impatient” and altogether unsympathetic. However, the findings were in line with those expressed by [3] in a cross-sectional hospital-based study involving 180 in-patients, conducted in India, which found that, overall, most of the clients were satisfied with the competence of the hospital staff.

The results also depicted nurses to be confidential in their dealings with respondents' personal information. The study found out that nurses kept personal information of patients well. These findings were in line with those expressed by [10] where patients for the most part in the study trusted that the nursing staff will maintain their dignity, privacy, and confidentiality of information as well as trusting that the staff knew what they were doing. The findings in this study are however in contrast with those identified by [21] involving six people who had experienced dissatisfaction during a hospital care episode; participants said they were treated disrespectfully, and their integrity was threatened and violated not only verbally but also physically. The results are also in contrast with those experienced by [10, 20] where the authors reported some lack of privacy with patients complaining that they had been overheard by people while discussing life-threatening issues as well as very confidential information.

The findings also showed that the respondents were generally satisfied with the nurses' ability to maintain a therapeutic work environment. The respondents claim nurses ensured that the wards were generally clean and in good shape for patients even though some claim their washrooms were not in good shape. On the whole, respondents seem satisfied with nurses' maintenance of a therapeutic work environment. These findings are in line with those expressed by [3] where the organizational environment of the hospital was seen to be therapeutic. The researchers claim in their study the satisfaction of patients with the organizational arrangement in Yemen Central Hospitals included cleanliness of the environment, nutritious food, and low noise in the wards in particular.

On the topic of effective communication of nurses, most of the respondents claim nurses were very unprofessional when communicating with patients. Nurses were said to have communicated harshly with patients and their relatives and in fact made them very dissatisfied. They said nurses were very rude and unsympathetic to them and their relatives when providing nursing care. The finding was in line with those expressed by [3, 21] where the patients reported nursing care providers who were “rude” and “snotty,” nurses who “blew patients off,” and staff who were “impatient” and altogether unsympathetic to client relations. The result is also in line with the findings by [19] where patients reported that they were misunderstood or not taken seriously because of one-way communication and that the communication they received was delivered in a technical language that was hard to understand and eventually led to dissatisfaction with nursing care. The findings are also in line with those expressed by [3] where surgical patients stated that they were least satisfied with provision of information to them especially before surgery, but in contrast with the findings by [11] where patients reported information/communication was given in a thoughtful way in a quiet area, which was appreciated by the concerned patients.

The study established that the nurses did not provide sufficient and effective health education tailored towards meeting the needs of the patients. Patients claim nurses never had adequate time to educate them on their conditions. Even on the few occasions in which they were given education, they were rushed by the nurses and the language used was too technical. These findings are in line with those expressed by [19] where clients reported that sometimes they were misunderstood or not taken seriously because of one-way communication and that the communication they received was delivered in a technical language that was hard to understand and eventually led to dissatisfaction with nursing care.

Finally, on the satisfaction of clients with the professional behaviour of nurses and their competence in delivering nursing care, most of the respondents found nurses to be highly trained with key competence at providing effective nursing care. They stated that nurses showed dexterity in their rendering of care. These findings are in contrast with those identified by [7, 11] who conducted a quantitative study to explore patients' perceptions of the quality of nursing care. They found, using descriptive statistics to analyze patients' data, that the overall mean score was low with regard to the professional attitudes of nurses. However, the findings are in line with those identified by [20] who noted that type of food served, competence level of nursing staff, responsiveness of nurses to patients' needs, and access to fresh air/therapeutic environment rather than remaining in an air-conditioned room were identified as key factors that influenced patients' satisfaction with nursing care as well as decision to return to a facility.

Conclusively, respondents expressed satisfaction with nurses maintaining their confidentiality, maintaining a therapeutic environment, and being competent and professional in their rendering of nursing care. There was however dissatisfaction in the timely responsiveness of nurses to the needs of the respondents and prompt pain management, effective communication, and an efficient health education. It is thus necessary for nurse managers and the institution to re-orient nursing staff in these key areas of dissatisfaction.

### 5.3. Possible Factors Limiting the Achievement of Expectations of Nursing Care

On factors limiting the achievement of patients' expectations, the results revealed that disproportionate distribution of staff especially during the night duties resulted in high workload for the night nurses making them not meet the expectations of the respondents. The nurses especially on the wards during night duties were overburdened making them unable to meet all the needs of patients. The respondents further claim the hospital being a teaching and research facility placed a lot of demands on it making its workers overburdened especially at night where few staff were allotted without students. This finding is in line with those by [10] where it is reported that hospital context influenced the expectations of patients. In their study, patients complained that the size of the hospital also led to different patient expectations and perceptions of care with patients expecting a higher quality of care in a private or small hospital as opposed to a larger public hospital. The respondents claim larger hospitals led to increased work load on nurses and hence reduced quality of care.

Low morale of nurses for the profession was also identified as another factor accounting for respondents' expectations not being met. They stated that most of the new nurses were not “caring enough” and only entered the profession because of the financial gains and thus did their work unprofessionally/unethically or without passion. This finding is in line with those expressed by [11] where it was stated that staff morale affected their commitment to work.

Inadequacy of working resources was identified also as another factor accounting for nurses' inability to meet their expectations. They claim there were instances that nurses could not help them because they had insufficient materials to work with. This finding is supported by those expressed by [19] who stated that the nonavailability of consumables and requisite equipment for work resulted in poor service delivery for respondents.

Prolonged stay was seen as another factor accounting for nurses' inability to meet their needs. According to the respondents, nurses generally failed to ensure their needs were met because of prolonged stay leading to overfamiliarity and hence neglect. This finding is in line with those expressed by [10, 20] where overfamiliarity could lead to neglect as staff concentrated on new staff and emergency cases.

The noninvolvement of the respondents in their care was listed as one of the factors accounting for their expectations not being met. They stated that nurses failed to involve them when rendering care and this eventually led to reduced performance and hence their expectations not being met. This finding is in line with those stated by [20] who stated that patient participation in the care enhances patient satisfaction. Patients' ability to participate and be actively involved in their care and decision-making has an influence on their perception of satisfaction [5]. The findings are also in line with those identified by [3] in which the subjects reported that almost all of them want to participate in their care to the extent that they want accurate, honest, and complete information about their illness, treatment options, and prognosis. In addition, patients want their relations to be involved in their care but to take secondary role in decision-making.

## 6. Summary

The study sought to explore the expectations of patients with hypertension on the nursing care prior to admission at the KBTH and satisfaction level of patients with nursing care and to find out the factors limiting the delivery of the expected nursing care by the nurses.

On the expectations of respondents with nursing care, it was realized that the respondents expected the following: prompt pain management and responsiveness of nurses to clients' needs, high confidentiality level of nurses with clients' information, provision of effective and understandable health education, maintaining a therapeutic environment, effective communication, and a competent, ethical, and professional practice of nursing.

The study found out that the respondents were satisfied with the competence of nurse and professional attitudes of nurses, maintenance of a therapeutic environment, and maintaining the confidentiality of clients' information. However, respondents expressed gross dissatisfaction with prompt pain management of nurses and responsiveness of nurses to patient needs, effectiveness of health education, and efficient communication.

On the factors accounting for patients expectations of nursing care not being met, it was identified that the following factors were responsible: disproportionate distribution of staff for all the shifts, low morale for nursing, inadequate working equipment, noninvolvement of patients in nursing care, prolonged stay in the facility, among others.

## 7. Recommendations

The findings provide some insights about how to improve care for hypertensives in particular and all patients in general. It is recommended that regular in-service training in the areas of dissatisfaction must be pursued in order to improve the nursing care given to patients visiting healthcare organizations. Continuous professional development programs should target the areas of dissatisfaction such as prompt pain management, effective and culturally sensitive communication, and the techniques of delivering effective health education. Also, continuous monitoring of nursing professionals through regular appraisal systems should be adhered to so as to ensure nurses practice ethically and professionally since unethical behaviour of some nurses was cited as one of the reasons for substandard nursing care.

The findings of this study, even though relating directly to hypertensives, could be applied to other patients. Thus, regulatory body for nursing, the Nursing and Midwifery Council, needs to ensure proper supervision of nursing students and nurses especially during training so that those without the right attitude and passion for nursing are taken out of the noble profession. Courses like communication skills, sociology, and psychology need to be taken seriously and should be incorporated into the licensing examinations of nursing students. These courses would help the student nurses to understand clients better and communicate effectively before leaving school to practice as nurses.

### 7.1. Suggestions for Further Research

The study could not produce a tool for measuring patients' satisfaction in the African context. It is suggested that further research should focus on producing a cultural specific tool for assessing patients' satisfaction in sub-Saharan populace.

## Data Availability

The data used to support the findings of this study are available from the corresponding author upon request.

## Abbreviations

CVD: Cardiovascular disease
GHS: Ghana health service
KBTH: Korle-Bu Teaching Hospital
NCD: Noncommunicable disease
OPD: Out-Patient Department
SSA: Sub-Saharan Africa
WHO: World Health Organization.
